# μ-4,4′-Bipyridine-κ^2^ *N*:*N*′-bis­[aqua­(4,4′-bi­pyridine-κ*N*)(l-valinato-κ^2^ *N*,*O*)copper(II)] dinitrate dihydrate

**DOI:** 10.1107/S1600536808002109

**Published:** 2008-01-25

**Authors:** Ben-Yong Lou, Mao-Chun Hong

**Affiliations:** a Department of Chemistry and Chemical Engineering, Minjiang University, Fuzhou 350108, People’s Republic of China; b State Key Laboratory of Structural Chemistry, FuJian Institute of Research on the Structure of Matter, Fuzhou 350002, People’s Republic of China

## Abstract

In the title dinuclear complex, [Cu_2_(C_5_H_10_NO_2_)_2_(C_10_H_8_N_2_)_3_(H_2_O)_2_](NO_3_)_2_·2H_2_O, each of the two l-valinate anions chelates a Cu^II^ center through the amino N and carboxyl­ate O atom, forming a five-membered ring. A 4,4′-bipyridine mol­ecule bridges two water-coordinated Cu atoms, each of which is connected to another 4,4′-bipyridine, giving rise to a square-pyramidal coordination geometry for the Cu^II^ centers. The dinuclear dications, nitrate anions and uncoord­inated water mol­ecules are linked into a two-dimensional structure.

## Related literature

For background, see: Yamauchi *et al.* (2002 ▶).
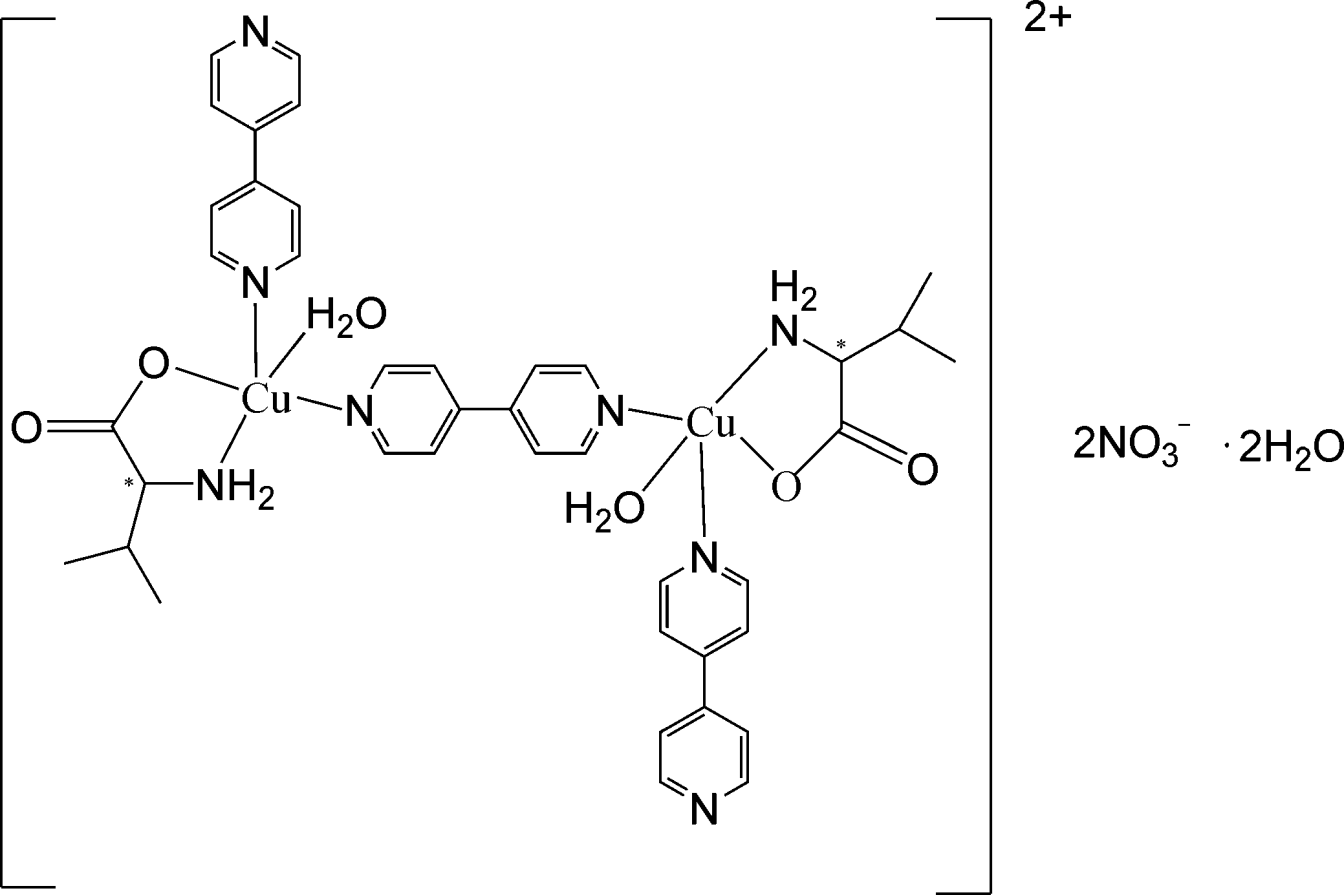


## Experimental

### 

#### Crystal data

[Cu_2_(C_5_H_10_NO_2_)_2_(C_10_H_8_N_2_)_3_(H_2_O)_2_](NO_3_)_2_·2H_2_O
*M* *_r_* = 1024.00
Triclinic, 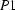

*a* = 8.9675 (14) Å
*b* = 9.6545 (16) Å
*c* = 13.9421 (15) Å
α = 91.533 (5)°
β = 100.384 (4)°
γ = 105.393 (8)°
*V* = 1141.2 (3) Å^3^
*Z* = 1
Mo *K*α radiation
μ = 1.01 mm^−1^
*T* = 293 (2) K
0.20 × 0.15 × 0.13 mm

#### Data collection

Rigaku Mercury CCD diffractometer
Absorption correction: multi-scan (*CrystalClear*; Rigaku, 2000 ▶) *T*_min_ = 0.824, *T*_max_ = 0.880
8898 measured reflections
6761 independent reflections
5710 reflections with *I* > 2σ(*I*)
*R*_int_ = 0.017

#### Refinement

*R*[*F* ^2^ > 2σ(*F* ^2^)] = 0.034
*wR*(*F* ^2^) = 0.086
*S* = 1.02
6761 reflections
599 parameters
3 restraints
H-atom parameters constrained
Δρ_max_ = 0.38 e Å^−3^
Δρ_min_ = −0.34 e Å^−3^
Absolute structure: Flack (1983 ▶), 1599 Friedel pairs
Flack parameter: 0.006 (12)

### 

Data collection: *CrystalClear* (Rigaku, 2000 ▶); cell refinement: *CrystalClear*; data reduction: *CrystalClear*; program(s) used to solve structure: *SHELXS97* (Sheldrick, 2008 ▶); program(s) used to refine structure: *SHELXL97* (Sheldrick, 2008 ▶); molecular graphics: *X-SEED* (Barbour, 2001 ▶); software used to prepare material for publication: *SHELXL97*.

## Supplementary Material

Crystal structure: contains datablocks I, global. DOI: 10.1107/S1600536808002109/ng2423sup1.cif

Structure factors: contains datablocks I. DOI: 10.1107/S1600536808002109/ng2423Isup2.hkl

Additional supplementary materials:  crystallographic information; 3D view; checkCIF report

## Figures and Tables

**Table:** 

Cu1—O1	1.937 (5)
Cu1—N4	1.993 (5)
Cu1—N1	2.011 (5)
Cu1—N5	2.031 (5)
Cu1—O3	2.275 (4)
Cu2—O4	1.944 (5)
Cu2—N2	1.967 (5)
Cu2—N3	2.001 (5)
Cu2—N7	2.028 (5)
Cu2—O6	2.308 (4)

**Table:** 

O1—Cu1—N4	172.71 (19)
O1—Cu1—N1	83.15 (19)
N4—Cu1—N1	95.7 (2)
O1—Cu1—N5	88.54 (19)
N4—Cu1—N5	90.7 (2)
N1—Cu1—N5	162.53 (18)
O1—Cu1—O3	92.32 (18)
N4—Cu1—O3	94.97 (18)
N1—Cu1—O3	98.71 (17)
N5—Cu1—O3	96.94 (17)
O4—Cu2—N2	84.0 (2)
O4—Cu2—N3	171.74 (19)
N2—Cu2—N3	95.2 (2)
O4—Cu2—N7	89.66 (19)
N2—Cu2—N7	165.8 (2)
N3—Cu2—N7	89.2 (2)
O4—Cu2—O6	92.17 (18)
N2—Cu2—O6	95.07 (18)
N3—Cu2—O6	96.10 (18)
N7—Cu2—O6	97.82 (17)

**Fig. 1.**
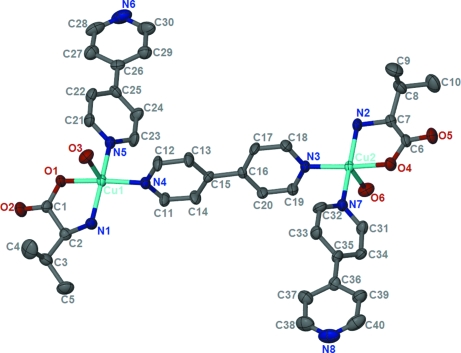
ORTEP of complex (I) with 50% thermal ellipsoids. Anions and solvent water were omitted.

**Fig. 2.**
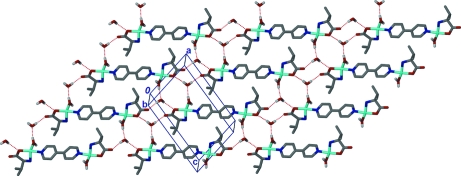
two-dimensional hydrogen-bonded structure in (I). Terminal 4,4'-bipyridine molecules were omitted.

**Fig. 3.**
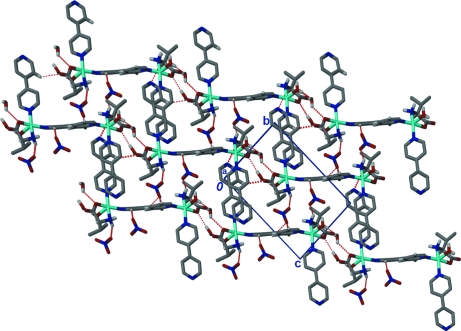
The packing structure viewed along a axis.

## supplementary crystallographic information

### Comment

Metal–amino acid complexes have been attracting considerable interests due to their structural feature and biological relevance (Yamauchi *et al.*, 2002). In the contribution, we report the title binuclear complex (I) in which there exist various hydrogen-bonding interactions cooperatively engineering the binuclear unit into ordered supramolecular structure.

In the structure of (I), deprotonated *L*-valine chelates Cu^II^ center through amino N and carboxylic O to form a five-membered ring of Cu^II^-amino acid. One bridging 4,4'-bipyridine molecule connects two Cu^II^-amino acid units into a chiral cation binuclear complex and two terminal 4,4'-bipyridine and two water molecules complete the square-pyramidal coordination geometry of Cu^II^ center (Fig1). Nitrate anion as H-bonded acceptors is simultaneously hydrogen-bonded to amino N and coordinated water (O3—H3A···O9; N1—H1B···O8; O6—H6A···O10; N2—H2A··· O12; Table 2). Solvent water molecule is simultaneously hydrogen-bonded to coordinated water and two symmetry-related carboxylic groups (O6—H6B···O13; O13—H13A···O5; O13—H13B···O1; O14—H14A···O4; O14—H14B···O2; O3—H3B···O14; Table 2). As a result, two solvent water, two coordinated water and two Cu^II^ -amino acid unit form a supramolecular synthon *R*_4_^4^(12). And two solvent water and two carboxylic groups form another synthon *R*_4_^4^(12). The two synthons connect the binuclear unit parallel to each other into a two-dimensional structure (Fig2). Moreover, two deprotonated *L*-valine in the binuclear unit are involved in different weak hydrogen-bonding interactions with terminal 4,4'- bipyridine. One interacts with 4,4'-bipyridine through C—H···N interactions between the C—H group of *L*-valine and N atom of 4,4'-bipyridine (C2—H2···N8). And the other is involved in C—H···O interactions with 4,4'-bipyridine between carboxylic O atom of *L*-valine and C—H group of 4,4'-bipyridine (C37—H37 ···O5). The bridging 4,4'-bipyridine is also involved in C—H···O interactions with two nitrate anions (C11—H11···O11; C13—H13···O11; C17—H17··· O11; C14—H14···O7; C18—H18···O7). The C—H···O(N) interactions connect the layers into ordered packing structure (Fig. 3).

### Experimental

To an aqueous solution (10 ml) of *L*-valine (29 mg, 0.25 mmol) and NaOH (10 mg, 0.25 mmol), Cu(NO_3_)_2_.3H_2_O (60 mg, 0.25 mmol) in water (10 ml) was added slowly. The reaction solution was stirred for half an hour and then 4,4'-bipyridine (39 mg, 0.25 mmol) in ethanol (5 ml) was added. The solution was kept in air and after several days blue crystals were obtained.

### Refinement

H atoms bonded to C or N were located geometrically (C—H = 0.95–1.00 Å, N—H = 0.92 Å) with *U*_iso_(H) = 1.2 *U*_eq_(C,*N*) or 1.5 *U*_eq_(C). H atoms bonded to O were located by difference maps and constrained to ride on their parent atoms with *U*_iso_(H) = 1.2 *U*_eq_(O).

### Crystal data

**Table:**

[Cu_2_(C_5_H_10_NO_2_)_2_(C_10_H_8_N_2_)_3_(H_2_O)_2_](NO_3_)_2_·2H_2_O	*Z* = 1
*M**_r_* = 1024.00	*F*_000_ = 532
Triclinic, *P*1	*D*_x_ = 1.490 Mg m^−^^3^
Hall symbol: P 1	Mo *K*α radiation λ = 0.71073 Å
*a* = 8.9675 (14) Å	Cell parameters from 3138 reflections
*b* = 9.6545 (16) Å	θ = 3.0–27.5º
*c* = 13.9421 (15) Å	µ = 1.01 mm^−^^1^
α = 91.533 (5)º	*T* = 293 (2) K
β = 100.384 (4)º	Prism, blue
γ = 105.393 (8)º	0.20 × 0.15 × 0.13 mm
*V* = 1141.2 (3) Å^3^	

### Data collection

**Table:**

Rigaku Mercury CCD diffractometer	6761 independent reflections
Radiation source: fine-focus sealed tube	5710 reflections with *I* > 2σ(*I*)
Monochromator: graphite	*R*_int_ = 0.017
Detector resolution: 14.6306 pixels mm^-1^	θ_max_ = 27.5º
*T* = 293(2) K	θ_min_ = 3.0º
CCD scans	*h* = −11→10
Absorption correction: multi-scan(CrystalClear; Rigaku, 2000)	*k* = −12→11
*T*_min_ = 0.824, *T*_max_ = 0.880	*l* = −18→18
8898 measured reflections	

### Refinement

**Table:**

Refinement on *F*^2^	Hydrogen site location: inferred from neighbouring sites
Least-squares matrix: full	H-atom parameters constrained
*R*[*F*^2^ > 2σ(*F*^2^)] = 0.034	*w* = 1/[σ^2^(*F*_o_^2^) + (0.0466*P*)^2^] where *P* = (*F*_o_^2^ + 2*F*_c_^2^)/3
*wR*(*F*^2^) = 0.086	(Δ/σ)_max_ = 0.001
*S* = 1.02	Δρ_max_ = 0.38 e Å^−^^3^
6761 reflections	Δρ_min_ = −0.33 e Å^−^^3^
599 parameters	Extinction correction: none
3 restraints	Absolute structure: Flack (1983), 1599 Friedel pairs
Primary atom site location: structure-invariant direct methods	Flack parameter: 0.006 (12)
Secondary atom site location: difference Fourier map	

### Special details

**Table:**

Geometry. All e.s.d.'s (except the e.s.d. in the dihedral angle between two l.s. planes) are estimated using the full covariance matrix. The cell e.s.d.'s are taken into account individually in the estimation of e.s.d.'s in distances, angles and torsion angles; correlations between e.s.d.'s in cell parameters are only used when they are defined by crystal symmetry. An approximate (isotropic) treatment of cell e.s.d.'s is used for estimating e.s.d.'s involving l.s. planes.
Refinement. Refinement of *F*^2^ against ALL reflections. The weighted *R*-factor *wR* and goodness of fit *S* are based on *F*^2^, conventional *R*-factors *R* are based on *F*, with *F* set to zero for negative *F*^2^. The threshold expression of *F*^2^ > σ(*F*^2^) is used only for calculating *R*-factors(gt) *etc*. and is not relevant to the choice of reflections for refinement. *R*-factors based on *F*^2^ are statistically about twice as large as those based on *F*, and *R*- factors based on ALL data will be even larger.

### Fractional atomic coordinates and isotropic or equivalent isotropic displacement parameters (Å^2^)

**Table:**

	*x*	*y*	*z*	*U*_iso_*/*U*_eq_	
Cu1	1.44365 (4)	1.34168 (4)	0.93159 (3)	0.03451 (16)	
Cu2	0.46068 (4)	0.60988 (4)	0.41250 (3)	0.03179 (14)	
O1	1.6220 (5)	1.4275 (5)	1.0358 (3)	0.0414 (10)	
O2	1.8726 (5)	1.5493 (5)	1.0699 (3)	0.0547 (10)	
O3	1.3283 (6)	1.5205 (4)	0.9533 (3)	0.0489 (11)	
H3A	1.3230	1.5799	0.9068	0.079*	
H3B	1.2775	1.5435	0.9963	0.079*	
O4	0.2748 (5)	0.5222 (5)	0.3128 (3)	0.0401 (10)	
O5	0.0406 (5)	0.3648 (5)	0.2886 (3)	0.0562 (11)	
O6	0.5689 (6)	0.4220 (5)	0.3910 (3)	0.0513 (11)	
H6A	0.5718	0.3662	0.4372	0.079*	
H6B	0.6061	0.4008	0.3420	0.079*	
O7	0.2765 (6)	0.7359 (5)	0.6583 (3)	0.0569 (13)	
O8	0.3912 (11)	0.5947 (8)	0.7363 (5)	0.128 (3)	
O9	0.3206 (10)	0.7456 (7)	0.8172 (4)	0.105 (3)	
O10	0.5740 (10)	0.1982 (7)	0.5340 (4)	0.103 (2)	
O11	0.6212 (7)	0.2018 (6)	0.6897 (4)	0.0755 (16)	
O12	0.5171 (11)	0.3504 (9)	0.6218 (5)	0.133 (3)	
O13	0.7221 (6)	0.3668 (6)	0.2437 (3)	0.0617 (12)	
H13A	0.8153	0.3583	0.2568	0.079*	
H13B	0.6996	0.3801	0.1821	0.079*	
O14	0.1903 (6)	0.5750 (6)	0.1060 (3)	0.0594 (12)	
H14B	0.0888	0.5568	0.0890	0.079*	
H14A	0.1924	0.5122	0.1521	0.079*	
N1	1.5915 (6)	1.4398 (5)	0.8460 (3)	0.0375 (10)	
H1A	1.5804	1.3805	0.7909	0.045*	
H1B	1.5685	1.5234	0.8267	0.045*	
N2	0.3291 (6)	0.5138 (5)	0.5031 (3)	0.0387 (11)	
H2A	0.3902	0.4837	0.5541	0.046*	
H2B	0.2825	0.5769	0.5284	0.046*	
N3	0.6375 (6)	0.7231 (5)	0.5173 (4)	0.0322 (10)	
N4	1.2736 (6)	1.2338 (5)	0.8221 (4)	0.0331 (10)	
N5	1.3457 (6)	1.2108 (5)	1.0285 (3)	0.0365 (11)	
N6	1.1150 (9)	0.7765 (7)	1.4081 (4)	0.0727 (18)	
N7	0.5556 (6)	0.7360 (5)	0.3126 (3)	0.0357 (11)	
N8	0.8214 (9)	1.1567 (7)	−0.0611 (5)	0.0682 (17)	
N9	0.3331 (8)	0.6965 (6)	0.7384 (4)	0.0563 (15)	
N10	0.5720 (8)	0.2485 (6)	0.6131 (4)	0.0542 (15)	
C1	1.7529 (7)	1.4877 (5)	1.0111 (4)	0.0347 (11)	
C2	1.7546 (4)	1.4731 (4)	0.9019 (3)	0.0356 (8)	
H2	1.7908	1.3854	0.8914	0.043*	
C3	1.8748 (6)	1.5980 (6)	0.8693 (4)	0.0443 (12)	
H3	1.9803	1.6063	0.9111	0.053*	
C4	1.8349 (8)	1.7429 (5)	0.8827 (4)	0.0742 (17)	
H4A	1.7400	1.7428	0.8351	0.111*	
H4B	1.9233	1.8222	0.8725	0.111*	
H4C	1.8161	1.7553	0.9491	0.111*	
C5	1.8858 (7)	1.5663 (5)	0.7628 (3)	0.0593 (12)	
H5A	1.9108	1.4741	0.7561	0.089*	
H5B	1.9689	1.6434	0.7443	0.089*	
H5C	1.7847	1.5612	0.7200	0.089*	
C6	0.1648 (6)	0.4266 (5)	0.3431 (4)	0.0354 (11)	
C7	0.2064 (5)	0.3883 (4)	0.4487 (3)	0.0373 (8)	
H7	0.2583	0.3090	0.4457	0.045*	
C8	0.0700 (6)	0.3324 (6)	0.5031 (4)	0.0520 (13)	
H8	0.1203	0.3210	0.5714	0.062*	
C9	−0.0243 (6)	0.4340 (6)	0.5120 (4)	0.0728 (15)	
H9A	0.0430	0.5229	0.5493	0.109*	
H9B	−0.1100	0.3905	0.5459	0.109*	
H9C	−0.0689	0.4561	0.4466	0.109*	
C10	−0.0322 (7)	0.1801 (6)	0.4606 (5)	0.0737 (17)	
H10A	−0.1177	0.1476	0.4968	0.111*	
H10B	0.0334	0.1130	0.4667	0.111*	
H10C	−0.0770	0.1833	0.3915	0.111*	
C11	1.2950 (8)	1.1217 (6)	0.7721 (4)	0.0415 (13)	
H11	1.3985	1.1104	0.7797	0.050*	
C12	1.1276 (7)	1.2440 (6)	0.8114 (4)	0.0425 (13)	
H12	1.1094	1.3214	0.8468	0.051*	
C13	0.9983 (7)	1.1477 (6)	0.7511 (4)	0.0434 (13)	
H13	0.8960	1.1612	0.7449	0.052*	
C14	1.1768 (7)	1.0244 (7)	0.7116 (5)	0.0421 (14)	
H14	1.1997	0.9496	0.6762	0.051*	
C15	1.0217 (7)	1.0321 (6)	0.7004 (4)	0.0307 (12)	
C16	0.8883 (6)	0.9223 (5)	0.6389 (4)	0.0273 (11)	
C17	0.7350 (7)	0.9183 (6)	0.6421 (4)	0.0414 (13)	
H17	0.7128	0.9855	0.6846	0.050*	
C18	0.6127 (7)	0.8150 (6)	0.5825 (4)	0.0401 (13)	
H18	0.5073	0.8094	0.5881	0.048*	
C19	0.7872 (7)	0.7270 (6)	0.5165 (4)	0.0403 (12)	
H19	0.8072	0.6605	0.4724	0.048*	
C20	0.9111 (7)	0.8201 (6)	0.5748 (4)	0.0356 (11)	
H20	1.0150	0.8161	0.5722	0.043*	
C21	1.3459 (8)	1.2699 (6)	1.1170 (4)	0.0429 (14)	
H21	1.3816	1.3718	1.1289	0.052*	
C22	1.2974 (8)	1.1905 (6)	1.1902 (4)	0.0420 (14)	
H22	1.2995	1.2377	1.2513	0.050*	
C23	1.2924 (8)	1.0656 (6)	1.0143 (4)	0.0463 (15)	
H23	1.2868	1.0210	0.9516	0.056*	
C24	1.2458 (9)	0.9790 (6)	1.0871 (4)	0.0473 (17)	
H24	1.2147	0.8771	1.0752	0.057*	
C25	1.2447 (7)	1.0414 (6)	1.1769 (4)	0.0363 (13)	
C26	1.1970 (8)	0.9493 (7)	1.2579 (4)	0.0400 (14)	
C27	1.2756 (10)	0.9897 (8)	1.3550 (5)	0.0521 (18)	
H27	1.3574	1.0770	1.3719	0.063*	
C28	1.2299 (10)	0.8980 (9)	1.4252 (5)	0.0626 (19)	
H28	1.2851	0.9242	1.4908	0.075*	
C29	1.0786 (9)	0.8260 (7)	1.2401 (5)	0.0475 (14)	
H29	1.0222	0.7969	1.1751	0.057*	
C30	1.0384 (10)	0.7417 (7)	1.3147 (6)	0.068 (2)	
H30	0.9537	0.6562	1.2997	0.082*	
C31	0.5625 (8)	0.6763 (6)	0.2275 (4)	0.0416 (14)	
H31	0.5320	0.5741	0.2179	0.050*	
C32	0.5987 (8)	0.8792 (6)	0.3249 (4)	0.0427 (14)	
H32	0.5925	0.9236	0.3851	0.051*	
C33	0.6518 (8)	0.9662 (6)	0.2545 (4)	0.0429 (15)	
H33	0.6847	1.0680	0.2672	0.051*	
C34	0.6119 (8)	0.7547 (6)	0.1514 (4)	0.0429 (14)	
H34	0.6147	0.7069	0.0915	0.052*	
C35	0.6568 (7)	0.9031 (6)	0.1641 (4)	0.0369 (13)	
C36	0.7132 (8)	0.9905 (6)	0.0870 (4)	0.0379 (13)	
C37	0.8484 (8)	1.1117 (7)	0.1097 (5)	0.0497 (15)	
H37	0.9043	1.1397	0.1750	0.060*	
C38	0.8942 (10)	1.1870 (7)	0.0314 (6)	0.0642 (18)	
H38	0.9855	1.2669	0.0456	0.077*	
C39	0.6370 (9)	0.9583 (7)	−0.0092 (5)	0.0487 (16)	
H39	0.5452	0.8793	−0.0266	0.058*	
C40	0.6957 (10)	1.0424 (8)	−0.0802 (5)	0.065 (2)	
H40	0.6433	1.0170	−0.1464	0.079*	

### Atomic displacement parameters (Å^2^)

**Table:**

	*U*^11^	*U*^22^	*U*^33^	*U*^12^	*U*^13^	*U*^23^
Cu1	0.0314 (4)	0.0371 (3)	0.0274 (3)	0.0003 (3)	0.0008 (3)	−0.0029 (3)
Cu2	0.0285 (3)	0.0338 (3)	0.0265 (3)	0.0000 (3)	0.0015 (3)	−0.0010 (2)
O1	0.034 (2)	0.049 (2)	0.030 (2)	−0.0082 (19)	0.0049 (18)	−0.0027 (18)
O2	0.0313 (19)	0.076 (3)	0.0424 (19)	−0.0012 (18)	−0.0050 (16)	−0.0032 (17)
O3	0.065 (3)	0.048 (2)	0.039 (2)	0.023 (2)	0.012 (2)	−0.0023 (18)
O4	0.033 (2)	0.050 (2)	0.030 (2)	0.005 (2)	−0.0026 (18)	0.0045 (18)
O5	0.035 (2)	0.067 (2)	0.050 (2)	−0.0029 (18)	−0.0056 (17)	−0.0101 (17)
O6	0.063 (3)	0.055 (3)	0.043 (2)	0.026 (2)	0.015 (2)	0.0064 (19)
O7	0.076 (4)	0.070 (3)	0.037 (2)	0.040 (3)	0.010 (2)	0.009 (2)
O8	0.188 (8)	0.127 (5)	0.092 (4)	0.110 (6)	−0.018 (4)	0.012 (4)
O9	0.157 (8)	0.111 (5)	0.051 (3)	0.043 (5)	0.024 (4)	−0.004 (3)
O10	0.141 (7)	0.109 (5)	0.055 (4)	0.029 (5)	0.018 (4)	−0.013 (3)
O11	0.077 (4)	0.085 (4)	0.079 (4)	0.043 (3)	0.020 (3)	0.029 (3)
O12	0.197 (9)	0.129 (5)	0.096 (4)	0.119 (6)	−0.026 (5)	0.005 (4)
O13	0.049 (3)	0.093 (3)	0.048 (2)	0.026 (3)	0.010 (2)	0.010 (2)
O14	0.050 (3)	0.101 (3)	0.036 (2)	0.033 (3)	0.014 (2)	0.008 (2)
N1	0.036 (2)	0.044 (3)	0.028 (2)	0.001 (2)	0.0062 (18)	0.0043 (18)
N2	0.033 (2)	0.043 (3)	0.030 (2)	−0.001 (2)	−0.0005 (18)	−0.0070 (19)
N3	0.032 (2)	0.031 (2)	0.028 (2)	0.0012 (19)	0.0028 (19)	−0.0043 (18)
N4	0.028 (2)	0.035 (2)	0.030 (2)	0.0024 (19)	0.0011 (19)	−0.0015 (19)
N5	0.035 (3)	0.035 (3)	0.034 (2)	0.003 (2)	0.004 (2)	−0.004 (2)
N6	0.111 (5)	0.066 (4)	0.057 (3)	0.033 (4)	0.044 (3)	0.018 (3)
N7	0.036 (3)	0.035 (3)	0.029 (2)	0.000 (2)	0.005 (2)	0.001 (2)
N8	0.097 (4)	0.055 (3)	0.075 (4)	0.035 (3)	0.049 (3)	0.034 (3)
N9	0.060 (4)	0.069 (4)	0.045 (3)	0.030 (3)	0.006 (3)	0.011 (3)
N10	0.057 (4)	0.054 (3)	0.049 (3)	0.013 (3)	0.007 (3)	0.005 (3)
C1	0.032 (2)	0.042 (3)	0.028 (2)	0.010 (2)	0.0005 (18)	−0.0002 (17)
C2	0.035 (2)	0.0362 (19)	0.0352 (18)	0.0093 (16)	0.0049 (16)	0.0037 (15)
C3	0.034 (3)	0.052 (3)	0.044 (3)	0.005 (2)	0.010 (2)	0.007 (2)
C4	0.098 (5)	0.038 (3)	0.079 (4)	0.006 (3)	0.015 (4)	0.012 (2)
C5	0.074 (3)	0.064 (3)	0.044 (2)	0.015 (3)	0.027 (2)	0.015 (2)
C6	0.026 (2)	0.036 (3)	0.039 (2)	0.0029 (19)	0.0010 (19)	−0.0030 (18)
C7	0.0327 (19)	0.0370 (19)	0.0382 (19)	0.0045 (16)	0.0049 (16)	−0.0011 (15)
C8	0.042 (3)	0.049 (3)	0.056 (3)	−0.006 (2)	0.015 (2)	0.006 (2)
C9	0.052 (3)	0.082 (4)	0.086 (4)	0.010 (3)	0.031 (3)	−0.001 (3)
C10	0.072 (4)	0.053 (3)	0.078 (4)	−0.013 (3)	0.013 (3)	0.008 (3)
C11	0.030 (3)	0.050 (3)	0.040 (3)	0.012 (3)	−0.004 (2)	−0.013 (2)
C12	0.037 (3)	0.033 (2)	0.050 (3)	0.008 (2)	−0.003 (2)	−0.018 (2)
C13	0.026 (2)	0.048 (3)	0.048 (3)	0.008 (2)	−0.006 (2)	−0.014 (2)
C14	0.022 (3)	0.048 (3)	0.053 (3)	0.010 (2)	0.000 (2)	−0.011 (2)
C15	0.035 (3)	0.035 (3)	0.021 (2)	0.010 (2)	0.004 (2)	−0.001 (2)
C16	0.026 (3)	0.023 (2)	0.029 (3)	0.002 (2)	0.003 (2)	0.001 (2)
C17	0.036 (3)	0.048 (3)	0.037 (3)	0.011 (3)	0.006 (2)	−0.022 (2)
C18	0.029 (3)	0.046 (3)	0.043 (3)	0.005 (2)	0.008 (2)	−0.003 (2)
C19	0.031 (3)	0.044 (3)	0.041 (2)	0.004 (2)	0.007 (2)	−0.009 (2)
C20	0.030 (2)	0.036 (2)	0.040 (2)	0.008 (2)	0.010 (2)	−0.0094 (19)
C21	0.048 (4)	0.033 (3)	0.039 (3)	−0.003 (3)	0.009 (3)	−0.008 (2)
C22	0.053 (4)	0.036 (3)	0.033 (3)	0.010 (3)	0.003 (3)	−0.007 (2)
C23	0.061 (4)	0.028 (3)	0.040 (3)	−0.001 (3)	0.006 (3)	−0.010 (2)
C24	0.072 (5)	0.030 (3)	0.040 (3)	0.009 (3)	0.019 (3)	0.000 (3)
C25	0.029 (3)	0.030 (3)	0.048 (3)	0.002 (2)	0.010 (3)	0.005 (2)
C26	0.042 (3)	0.045 (3)	0.039 (3)	0.016 (3)	0.018 (3)	0.005 (2)
C27	0.055 (4)	0.061 (4)	0.042 (4)	0.020 (4)	0.006 (3)	0.002 (3)
C28	0.084 (5)	0.084 (5)	0.036 (3)	0.043 (4)	0.021 (3)	0.012 (3)
C29	0.053 (3)	0.046 (3)	0.044 (3)	0.010 (3)	0.015 (3)	0.004 (2)
C30	0.093 (5)	0.042 (3)	0.077 (4)	0.009 (3)	0.049 (4)	0.008 (3)
C31	0.052 (4)	0.031 (3)	0.037 (3)	0.006 (3)	0.005 (3)	0.000 (2)
C32	0.056 (4)	0.046 (3)	0.030 (3)	0.014 (3)	0.018 (3)	0.002 (2)
C33	0.052 (4)	0.030 (3)	0.041 (3)	0.002 (3)	0.010 (3)	−0.003 (3)
C34	0.055 (4)	0.035 (3)	0.036 (3)	0.001 (3)	0.020 (3)	−0.007 (2)
C35	0.037 (3)	0.046 (3)	0.024 (2)	0.009 (3)	0.000 (2)	−0.001 (2)
C36	0.048 (4)	0.027 (3)	0.038 (3)	0.011 (2)	0.006 (3)	0.003 (2)
C37	0.046 (3)	0.043 (3)	0.060 (4)	0.010 (3)	0.015 (3)	0.010 (3)
C38	0.078 (4)	0.047 (3)	0.074 (4)	0.014 (3)	0.033 (4)	0.019 (3)
C39	0.060 (5)	0.050 (4)	0.038 (3)	0.014 (3)	0.015 (3)	0.004 (3)
C40	0.107 (6)	0.063 (4)	0.042 (3)	0.044 (4)	0.023 (4)	0.014 (3)

### Geometric parameters (Å, °)

**Table:**

Cu1—O1	1.937 (5)	C8—C9	1.471 (7)
Cu1—N4	1.993 (5)	C8—C10	1.548 (8)
Cu1—N1	2.011 (5)	C8—H8	1.0000
Cu1—N5	2.031 (5)	C9—H9A	0.9800
Cu1—O3	2.275 (4)	C9—H9B	0.9800
Cu2—O4	1.944 (5)	C9—H9C	0.9800
Cu2—N2	1.967 (5)	C10—H10A	0.9800
Cu2—N3	2.001 (5)	C10—H10B	0.9800
Cu2—N7	2.028 (5)	C10—H10C	0.9800
Cu2—O6	2.308 (4)	C11—C14	1.351 (9)
O1—C1	1.276 (7)	C11—H11	0.9500
O2—C1	1.223 (7)	C12—C13	1.396 (8)
O3—H3A	0.8807	C12—H12	0.9500
O3—H3B	0.8719	C13—C15	1.388 (7)
O4—C6	1.300 (7)	C13—H13	0.9500
O5—C6	1.221 (7)	C14—C15	1.393 (8)
O6—H6A	0.8522	C14—H14	0.9500
O6—H6B	0.8556	C15—C16	1.483 (4)
O7—N9	1.252 (6)	C16—C17	1.374 (8)
O8—N9	1.230 (7)	C16—C20	1.393 (7)
O9—N9	1.220 (7)	C17—C18	1.391 (9)
O10—N10	1.198 (7)	C17—H17	0.9500
O11—N10	1.224 (7)	C18—H18	0.9500
O12—N10	1.224 (7)	C19—C20	1.344 (8)
O13—H13A	0.8487	C19—H19	0.9500
O13—H13B	0.8665	C20—H20	0.9500
O14—H14B	0.8663	C21—C22	1.357 (8)
O14—H14A	0.8976	C21—H21	0.9500
N1—C2	1.475 (6)	C22—C25	1.387 (8)
N1—H1A	0.9200	C22—H22	0.9500
N1—H1B	0.9200	C23—C24	1.384 (8)
N2—C7	1.481 (6)	C23—H23	0.9500
N2—H2A	0.9200	C24—C25	1.377 (8)
N2—H2B	0.9200	C24—H24	0.9500
N3—C19	1.335 (8)	C25—C26	1.508 (7)
N3—C18	1.340 (7)	C26—C29	1.351 (9)
N4—C12	1.322 (8)	C26—C27	1.401 (9)
N4—C11	1.347 (7)	C27—C28	1.382 (10)
N5—C21	1.344 (7)	C27—H27	0.9500
N5—C23	1.353 (7)	C28—H28	0.9500
N6—C28	1.323 (10)	C29—C30	1.378 (9)
N6—C30	1.346 (10)	C29—H29	0.9500
N7—C31	1.325 (7)	C30—H30	0.9500
N7—C32	1.330 (7)	C31—C34	1.389 (8)
N8—C38	1.323 (10)	C31—H31	0.9500
N8—C40	1.333 (10)	C32—C33	1.377 (8)
C1—C2	1.529 (6)	C32—H32	0.9500
C2—C3	1.529 (6)	C33—C35	1.398 (8)
C2—H2	1.0000	C33—H33	0.9500
C3—C5	1.533 (7)	C34—C35	1.379 (8)
C3—C4	1.547 (7)	C34—H34	0.9500
C3—H3	1.0000	C35—C36	1.463 (7)
C4—H4A	0.9800	C36—C39	1.376 (8)
C4—H4B	0.9800	C36—C37	1.424 (9)
C4—H4C	0.9800	C37—C38	1.391 (9)
C5—H5A	0.9800	C37—H37	0.9500
C5—H5B	0.9800	C38—H38	0.9500
C5—H5C	0.9800	C39—C40	1.386 (9)
C6—C7	1.533 (6)	C39—H39	0.9500
C7—C8	1.539 (6)	C40—H40	0.9500
C7—H7	1.0000		
			
O1—Cu1—N4	172.71 (19)	C10—C8—H8	106.0
O1—Cu1—N1	83.15 (19)	C8—C9—H9A	109.5
N4—Cu1—N1	95.7 (2)	C8—C9—H9B	109.5
O1—Cu1—N5	88.54 (19)	H9A—C9—H9B	109.5
N4—Cu1—N5	90.7 (2)	C8—C9—H9C	109.5
N1—Cu1—N5	162.53 (18)	H9A—C9—H9C	109.5
O1—Cu1—O3	92.32 (18)	H9B—C9—H9C	109.5
N4—Cu1—O3	94.97 (18)	C8—C10—H10A	109.5
N1—Cu1—O3	98.71 (17)	C8—C10—H10B	109.5
N5—Cu1—O3	96.94 (17)	H10A—C10—H10B	109.5
O4—Cu2—N2	84.0 (2)	C8—C10—H10C	109.5
O4—Cu2—N3	171.74 (19)	H10A—C10—H10C	109.5
N2—Cu2—N3	95.2 (2)	H10B—C10—H10C	109.5
O4—Cu2—N7	89.66 (19)	N4—C11—C14	123.3 (6)
N2—Cu2—N7	165.8 (2)	N4—C11—H11	118.3
N3—Cu2—N7	89.2 (2)	C14—C11—H11	118.3
O4—Cu2—O6	92.17 (18)	N4—C12—C13	123.9 (5)
N2—Cu2—O6	95.07 (18)	N4—C12—H12	118.1
N3—Cu2—O6	96.10 (18)	C13—C12—H12	118.1
N7—Cu2—O6	97.82 (17)	C15—C13—C12	119.0 (5)
C1—O1—Cu1	117.2 (4)	C15—C13—H13	120.5
Cu1—O3—H3A	117.6	C12—C13—H13	120.5
Cu1—O3—H3B	135.4	C11—C14—C15	120.9 (5)
H3A—O3—H3B	106.8	C11—C14—H14	119.5
C6—O4—Cu2	114.9 (3)	C15—C14—H14	119.5
Cu2—O6—H6A	116.5	C13—C15—C14	116.2 (5)
Cu2—O6—H6B	128.1	C13—C15—C16	121.6 (4)
H6A—O6—H6B	115.4	C14—C15—C16	122.2 (4)
H13A—O13—H13B	108.7	C17—C16—C20	116.8 (5)
H14B—O14—H14A	97.6	C17—C16—C15	120.9 (4)
C2—N1—Cu1	108.7 (3)	C20—C16—C15	122.3 (4)
C2—N1—H1A	109.9	C16—C17—C18	119.4 (5)
Cu1—N1—H1A	109.9	C16—C17—H17	120.3
C2—N1—H1B	109.9	C18—C17—H17	120.3
Cu1—N1—H1B	109.9	N3—C18—C17	122.7 (6)
H1A—N1—H1B	108.3	N3—C18—H18	118.7
C7—N2—Cu2	108.5 (3)	C17—C18—H18	118.7
C7—N2—H2A	110.0	N3—C19—C20	123.5 (5)
Cu2—N2—H2A	110.0	N3—C19—H19	118.2
C7—N2—H2B	110.0	C20—C19—H19	118.2
Cu2—N2—H2B	110.0	C19—C20—C16	120.5 (5)
H2A—N2—H2B	108.4	C19—C20—H20	119.7
C19—N3—C18	116.9 (5)	C16—C20—H20	119.7
C19—N3—Cu2	121.5 (4)	N5—C21—C22	123.0 (5)
C18—N3—Cu2	120.8 (4)	N5—C21—H21	118.5
C12—N4—C11	116.6 (5)	C22—C21—H21	118.5
C12—N4—Cu1	122.1 (4)	C21—C22—C25	120.8 (5)
C11—N4—Cu1	119.3 (4)	C21—C22—H22	119.6
C21—N5—C23	116.7 (5)	C25—C22—H22	119.6
C21—N5—Cu1	118.3 (4)	N5—C23—C24	122.8 (5)
C23—N5—Cu1	124.7 (4)	N5—C23—H23	118.6
C28—N6—C30	116.4 (6)	C24—C23—H23	118.6
C31—N7—C32	117.4 (5)	C25—C24—C23	119.7 (5)
C31—N7—Cu2	119.6 (4)	C25—C24—H24	120.2
C32—N7—Cu2	122.6 (4)	C23—C24—H24	120.2
C38—N8—C40	116.7 (6)	C24—C25—C22	117.0 (5)
O9—N9—O8	118.8 (6)	C24—C25—C26	120.7 (5)
O9—N9—O7	123.0 (5)	C22—C25—C26	122.3 (5)
O8—N9—O7	117.8 (6)	C29—C26—C27	117.7 (6)
O10—N10—O12	121.0 (6)	C29—C26—C25	121.8 (6)
O10—N10—O11	123.4 (6)	C27—C26—C25	120.5 (6)
O12—N10—O11	115.6 (6)	C28—C27—C26	117.6 (7)
O2—C1—O1	123.3 (5)	C28—C27—H27	121.2
O2—C1—C2	120.9 (5)	C26—C27—H27	121.2
O1—C1—C2	115.8 (5)	N6—C28—C27	125.0 (7)
N1—C2—C1	109.2 (4)	N6—C28—H28	117.5
N1—C2—C3	116.2 (3)	C27—C28—H28	117.5
C1—C2—C3	113.1 (4)	C26—C29—C30	121.1 (7)
N1—C2—H2	105.8	C26—C29—H29	119.5
C1—C2—H2	105.8	C30—C29—H29	119.5
C3—C2—H2	105.8	N6—C30—C29	122.2 (7)
C2—C3—C5	110.2 (4)	N6—C30—H30	118.9
C2—C3—C4	111.7 (4)	C29—C30—H30	118.9
C5—C3—C4	111.0 (4)	N7—C31—C34	123.7 (5)
C2—C3—H3	107.9	N7—C31—H31	118.2
C5—C3—H3	107.9	C34—C31—H31	118.2
C4—C3—H3	107.9	N7—C32—C33	123.2 (5)
C3—C4—H4A	109.5	N7—C32—H32	118.4
C3—C4—H4B	109.5	C33—C32—H32	118.4
H4A—C4—H4B	109.5	C32—C33—C35	119.3 (5)
C3—C4—H4C	109.5	C32—C33—H33	120.4
H4A—C4—H4C	109.5	C35—C33—H33	120.4
H4B—C4—H4C	109.5	C35—C34—C31	118.9 (5)
C3—C5—H5A	109.5	C35—C34—H34	120.5
C3—C5—H5B	109.5	C31—C34—H34	120.5
H5A—C5—H5B	109.5	C34—C35—C33	117.4 (5)
C3—C5—H5C	109.5	C34—C35—C36	121.0 (5)
H5A—C5—H5C	109.5	C33—C35—C36	121.5 (5)
H5B—C5—H5C	109.5	C39—C36—C37	118.3 (5)
O5—C6—O4	121.9 (5)	C39—C36—C35	121.1 (6)
O5—C6—C7	122.3 (5)	C37—C36—C35	120.7 (5)
O4—C6—C7	115.7 (5)	C38—C37—C36	116.5 (7)
N2—C7—C6	107.5 (4)	C38—C37—H37	121.7
N2—C7—C8	112.4 (4)	C36—C37—H37	121.7
C6—C7—C8	117.7 (4)	N8—C38—C37	125.5 (7)
N2—C7—H7	106.1	N8—C38—H38	117.3
C6—C7—H7	106.1	C37—C38—H38	117.3
C8—C7—H7	106.1	C36—C39—C40	119.3 (7)
C9—C8—C7	114.0 (4)	C36—C39—H39	120.4
C9—C8—C10	112.8 (5)	C40—C39—H39	120.4
C7—C8—C10	111.5 (4)	N8—C40—C39	123.8 (7)
C9—C8—H8	106.0	N8—C40—H40	118.1
C7—C8—H8	106.0	C39—C40—H40	118.1

### Hydrogen-bond geometry (Å, °)

**Table:**

*D*—H···*A*	*D*—H	H···*A*	*D*···*A*	*D*—H···*A*
N2—H2A···O12	0.92	2.07	2.936 (7)	156
O6—H6A···O10	0.85	2.14	2.983 (7)	170
O6—H6B···O13	0.86	1.93	2.782 (6)	171
O14—H14A···O4	0.90	2.22	2.941 (6)	137
O14—H14B···O2^i^	0.87	1.89	2.745 (7)	168
O13—H13A···O5^ii^	0.85	1.97	2.816 (7)	173
O13—H13B···O1^iii^	0.87	2.14	2.997 (6)	172
N1—H1B···O8^iv^	0.92	2.12	2.892 (7)	141
O3—H3A···O9^iv^	0.88	2.06	2.930 (7)	170
O3—H3B···O14^v^	0.87	1.89	2.752 (6)	169

Symmetry codes: (i) *x*−2, *y*−1, *z*−1; (ii) *x*+1, *y*, *z*; (iii) *x*−1, *y*−1, *z*−1; (iv) *x*+1, *y*+1, *z*; (v) *x*+1, *y*+1, *z*+1.
